# Supplementary material for: Chromosome Ordinal Number-Related Genomic Stability Revealed Among Oryza and Other Poaceae Plants
Source: Int J Mol Sci. 2025 May 16;26(10):4778. doi: 10.3390/ijms26104778 (PMC12111954; doi:10.3390/ijms26104778)
Supplement: Supplementary file 1 [file ijms-26-04778-s001.zip › ijms-3571851-supplementary.pdf]

Supplementary tables

Supplementary Table S1. Homologous gene blocks and homologous genes in other species genome.

Species	Whole genome length	Number of tandem gene	Tandem/Million base pair
P. americarum	12.14	2562	211
S. italica	15.26	11114	728
S. bicolor	19.23	21833	1135

Supplementary Table S2. Homologous gene blocks and homologous genes between *L. perrieri* and other species.

Species	Gene number	Block	Gene/Block
P. americarum	13619	1112	12.25
S. italica	17104	1060	16.14
S. bicolor	16779	1220	13.75

Supplementary Table S3. Contents of tandem repeat genes in other species.

Species	Whole genome length	Number of tandem gene	Tandem/Million base pair
P. americarum	12.14	2562	211
S. italica	15.26	11114	728
S. bicolor	19.23	21833	1135

Supplementary Table S4. The proportion of tandem repeat genes on chromosomes

species	chr_number									
L. perrieri	3 and 10	7 and 9	1	2	11	4	8	12	5	6
S. bicolor	1	2	3	4	5	6	7	8	9	10
S. bicolor	0.469	0.300	0.486	0.359	0.721	0.609	0.374	0.400	0.592	0.493

Supplementary Table S5. The number of genes lost on the chromosomes of other species using *L. perrieri* as a reference. The number in the first row represents the chromosome ordinal number in each plant.

Species	1	2	3	4	5	6	7	8	9	10	11	12
P. americarum	1642	2090	2351	1216	2187	2356	1774					
S. italica	1410	1670	1491	1689	2976	1287	1856	1023	3702			
S. bicolor	2683	2122	2036	2128	652	1834	1396	970	1980	998		

Supplementary Table S6. Chromosome gene loss rates of other species with *L. perrieri* as reference. The chromosomes or chromosomal regions in each plant are mapped to their respective orthologous chromsomes in *L. perrieri*.

Species	1	2	3	4	5	6	7	8	9	10	11	12
S. bicolor	0.66	0.65	0.68	0.59	0.8	0.56	0.57	0.66	0.47	0.75		
S. italica	0.69	0.68	0.69	0.52	0.48	0.56	0.53	0.64	0.48			
P. americarum	0.72	0.65	0.61	0.7	0.53	0.54	0.56					

Supplementary Table S7 Genomic data information

Latin name	Abbreviation	Gene number	Whole genome length/Mbp	Genome Database Source
L. perrieri	Lp	29114	17.34	https://www.gramene.org
Minghui 63	Mh	57129	25.66	https://www.ncbi.nlm.nih.gov
O. barthii	Oa	31635	12.07	https://www.gramene.org
O. brachyantha	Ob	34588	11.30	https://www.gramene.org
O. glaberrima	Ol	35796	20.29	https://www.gramene.org
O. glumaepatula	Ou	37113	19.31	https://www.gramene.org
O. meridionalis	Om	29442	18.36	https://www.gramene.org
O. nivara	On	36379	16.81	https://www.gramene.org
O. punctata	Op	31791	17.79	https://www.gramene.org
O. rufipogon	Or	33163	19.59	https://www.gramene.org
O. sativa	Oj	40701	13.98	https://www.gramene.org
P. americarum	Pa	35791	12.14	https://cegresources.icrisat.org
S. bicolor	Sb	47005	19.23	https://www.gramene.org
S. italica	Si	40702	15.26	https://www.gramene.org
